# Comparative Retrospective Study of Tension-Free Vaginal Mesh Surgery, Native Tissue Repair, and Laparoscopic Sacrocolpopexy for Pelvic Organ Prolapse Repair

**DOI:** 10.1155/2020/7367403

**Published:** 2020-04-10

**Authors:** Haruhiko Kanasaki, Aki Oride, Tomomi Hara, Satoru Kyo

**Affiliations:** Department of Obstetrics and Gynecology, Faculty of Medicine, Shimane University, Izumo, Japan

## Abstract

**Methods:**

We identified that 308 women who had undergone surgical repair of POP were followed up for at least 6 months. Recurrence rates of POP after tension-free vaginal mesh (TVM) surgery (*n* = 243), native tissue repair (NTR) (vaginal hysterectomy with colpopexy, anterior and posterior colpoplasty, or circumferential suturing of the levator ani muscles and apical repair by transvaginal sacrospinous ligament fixation (SSLF)) (NTR; *n* = 31), and laparoscopic sacrocolpopexy after subtotal hysterectomy (LSC; *n* = 34) were compared. Presence of mesh erosion was also recorded.

**Results:**

Patients who underwent LSC were significantly younger (65.32 ± 3.23 years) than those who underwent TVM surgery (69.61 ± 8.31 years). After TVM surgery, the rate of recurrence (over POP-Q stage II) was 6.17% (15/243) and was highest in patients with advanced POP. The recurrence rate in patients who underwent NTR procedure was 3.23% (1/34) and that in patients who underwent LSC was 11.76% (4/11). There was no statistically significant difference in the recurrence rate between the three types of surgery. There were 13 cases (5.35%) of mesh erosion after TVM surgery and none after LSC surgery. The risk of mesh erosion was correlated with having had total TVM surgery but not with patient age or POP stage. Repeat procedures were performed in 5 women (2.14%) who underwent TVM surgery and 1 (2.94%) who underwent LSC. No patient underwent repeat surgery after NTR. There was no statistically significant difference in the reoperation rate between the three types of surgery.

**Conclusion:**

Our study suggested that TVM surgery, NTR, and LSC have comparable outcomes as for the postoperative recurrence rate and mesh erosion. However, the outcomes of each technique need to be carefully evaluated over a long period of time.

## 1. Introduction

Pelvic organ prolapse (POP) is a distressing health problem affecting about 15%–30% of women over 50 years of age [1]. The reported lifetime risk of surgery for POP ranges from 6% to 19% [2, 3]. Although a large number of surgical repair techniques for POP have been described, treatment of this condition continues to be a clinical challenge for urogynecological surgeons. Japanese gynecologists have traditionally performed vaginal hysterectomy (VH), anterior and posterior colpoplasty, or circumferential suturing of the levator ani muscles as a native tissue repair (NTR) procedure for POP. However, the pelvic floor structures become progressively weaker postoperatively in older women, contributing to a high rate of recurrence, especially in the anterior compartment [4]. Tension-free vaginal mesh (TVM) surgery for POP was first reported by French gynecologists in 2004 [5]. Thereafter, there was a rapid increase in transvaginal placement of synthetic mesh implants for POP in the belief that it was simpler to perform, less invasive, potentially able to preserve the uterus, and more effective than the traditional NTR [6]. Use of synthetic mesh increased by up to 40% in the USA [7]. However, in 2008 and again in 2011, the US Food and Drug Administration (FDA) expressed concern about the use of mesh for POP repair because of postoperative complications, including mesh exposure, mesh retraction, pain, and dyspareunia [8]. These FDA reports caused much controversy and raised ongoing questions about whether or not TVM surgery was appropriate for POP. Despite the number of randomized controlled trials that have investigated the use of mesh in female prolapse surgery, use of TVM remains controversial.

Abdominal sacrocolpopexy is widely used to repair apical vaginal prolapse but is not a popular method in Japan. However, with advances in laparoscopic surgery, it is now possible to perform laparoscopic sacrocolpopexy (LSC). The efficacy and safety of LSC for apical vaginal prolapse is now considered equivalent to that of ASC [9]. Furthermore, LSC is a uterus-sparing surgery, so it is becoming an increasingly common procedure [10] and has been reported to have a higher success rate and lower reoperation rate than TVM [11].

The first-line surgical procedure for POP is still not established because each of these three procedures has both advantages and disadvantages. In 2010, we started to perform TVM surgery in patients with POP rather than conventional NTR (VH, colpoplasty, or circumferential suturing of the levator ani muscles). However, after an FDA warning about transvaginal mesh in 2011 [12], we reverted to performing conventional NTR with addition of apical repair by transvaginal sacrospinous ligament fixation (SSLF). Furthermore, at the end of 2015, we started using LSC for POP repair, especially in relatively younger women. In this study, we retrospectively reviewed the outcomes, i.e., recurrence and mesh erosion, of these three types of POP surgery. The complications of TVM surgery were also evaluated, given that this was the most common procedure performed.

## 2. Materials and Methods

Data were collected retrospectively from medical records of patients who underwent POP surgery between January 2010 and May 2018 in the Department of Obstetrics and Gynecology at Shimane University Hospital after institutional review board approval and patient consent were obtained (20170224-1). In total, 308 women with preoperative POP quantification (POP-Q) over stage II were identified to have undergone prolapse repair (stage II; 68, stage III; 203, stage IV; 37). All patients were evaluated by physical examination with vaginal speculum in the decubitus position at rest and during a Valsalva maneuver, and then, TVM surgery, NTR (VH with anterior or posterior colpoplasty, circumferential suturing of the levator ani muscles, and SSLF as apical repair), or LSC was chosen according to patient age and background, POP-Q stage, and predominant descending part.

Conventional NTR and TVM surgery were performed under lumbar spinal anesthesia in the lithotomy position. VH, colpoplasty, and circumferential suturing of the levator ani muscles were performed in the usual fashion. SSLF was performed using a Capio™ SLIM device (Boston Scientific, Natick, MA) in all cases. A conventional TVM technique was used as described elsewhere [13]. However, because of the lack of availability of mesh kits for TVM surgery in Japan, we used monofilament polypropylene mesh (Polyform, Boston Scientific) cut into a shape similar to that used previously in the Prolift system (Ethicon, Somerville, NJ). TVM surgery included anterior TVM, posterior TVM, anterior and posterior TVM (TVM-AP), and total TVM (performed in patients with vaginal stump prolapse following hysterectomy). TVM with a modified shape of mesh with four arms was applied to the patients with a rectocele after hysterectomy (Enterocele-TVM; E-TVM). LSC was performed in the conventional manner [11] after subtotal hysterectomy. Polypropylene mesh (Polyform) which was manually cut into an appropriate shape was positioned in the anterior vesicovaginal space and secured to the anterior vaginal wall by absorbable #2-0 PolysorbTM (Covidien Japan, Tokyo, Japan) and uterine cervix by unabsorbable #0 Ti-Cron^TM^ (Covidien Japan, Tokyo, Japan). The mesh was then tightly fixed to the anterior longitudinal ligament at the sacral promontory by #0 Ti-CronTM. The mesh was not placed posteriorly in any of the patients who underwent LSC to avoid unexpected complications. Instead, posterior colpoplasty was applied if necessary.

All procedures were chosen and performed by either of two surgeons after examining the leading edge of the pelvic floor. The patients were discharged 3 days after surgery and monitored for complications in the outpatient clinic at 1, 3, 6, and 12 months postoperatively. Postoperative recurrence of POP was defined as POP-Q stage II or higher after the initial operation. One-way analysis of variance or the chi-squared test was used to test for statistically significant between-group differences in postoperative outcomes. A *P* value <0.05 was considered statistically significant.

## 3. Results

We performed 308 POP repairs between January 2010 and May 2018.  Table 1 shows the details and outcomes of the operations performed. During the study period, 243 patients (approximately 79%) underwent TVM surgery, 31 (10.1%) underwent NTR, and 34 (11.0%) underwent LSC. There was a statistically significant difference in mean age between the TVM group (69.61 ± 8.31 years) and the LSC group (65.32 ± 3.23 years) (*P* < 0.01). TVM surgery and LSC were mainly performed in women with POP-Q stage III and NTR in women with POP-Q stage II. During a minimum of 6 months of follow-up after the initial surgery, recurrence of POP, defined as POP-Q stage II or higher, occurred in 15 patients (6.17%) after TVM surgery, in 1 patients (3.23%) after NTR, and in 4 case (11.76%) after LSC surgeries. Mesh exposure was found in 13 (5.35%) of the 243 patients who underwent TVM surgery but not in any of the 34 patients who underwent LSC. There was no statistically significant difference in the recurrence rate or mesh exposure rate between the three types of surgery. A second operation was performed in 5 (2.14%) of the 243 women in the TVM surgery group because of postoperative complications. Although most of these complications were asymptomatic, 3 of the 5 women required a second operation due to continuous vaginal bleeding caused by mesh erosion and the remaining 2 underwent repeat TVM surgery because of subjective symptoms such as a sensation of dragging or difficulty voiding. A second operation (anterior and posterior TVM) was performed to treat POP recurrence in 1 of the 34 patients who underwent LSC. Only 1 of the 31 women who underwent NTR had postoperative POP recurrence, which is not presently serious, and she remains under follow-up.

The data for the 243 women who underwent TVM surgery were then reviewed in more detail.  Table 2 summarizes the data for patient age, POP-Q stage, and type of TVM surgery performed according to whether or not there was a recurrence during follow-up. Although the age range of these patients was wide, most were aged between 60 and 80 years. Recurrence of POP was observed in 9 (9.78%) of 92 women in their 60s, 1 (3.13%) of 32 in their 50s, and 5 (5.0%) of 100 in their 70s. The highest recurrence rate after TVM surgery was in the women in their 60s; however, there were no significant age-related differences in the recurrence rate (Table 2(a)). POP did not recur in any patient with POP-Q stage II, whereas recurrences were noted in 8 (4.77%) of 168 patients with stage POP-Q stage III and 7 (23.33%) of 30 patients with stage IV (Table 2(b)); the between-group differences were statistically significant (between POP-Q stage II and IV and between POP-Q stage III and IV). POP recurrence was observed in all types of TVM surgery performed and was particularly common in patients with POP-Q stage IV (Table 2(b)). Anterior and posterior TVM surgery was the most commonly performed TVM procedure and had a recurrence rate of 6.9% (10/145), which was similar to the overall recurrence rate for TVM surgery. There was no significant difference in the recurrence rate according to the type of TVM surgery performed (Table 2(c)) or in time since surgery (Table 2(d)). Recurrence was noticed as early as 3 months in some cases and as late as 3 years in others.

We then reviewed the patients in whom erosion of the mesh occurred after TVM surgery (Table 3). The mesh erosion rate was 50% (1/2) in women in their 40s, 12.5% (4/32) in those in their 50s, 1.1% (1/92) in those in their 60s, and 7.0% (7/100) in those in their 70s. Younger women tended to have a higher rate of mesh erosion, but there were no significant between-group age-related differences (Table 3(a)). The mesh erosion rate was 6.67% (3/45) in patients with POP-Q stage II and 4.17% (7/168) and 10.0% (3/30), respectively, in those with POP-Q stage III and IV. There was no statistically significant difference in the postoperative mesh exposure rate according to the POP-Q stage (Table 3(b)). However, when the mesh erosion rate was classified according to the type of TVM surgery, it was significantly higher in the women who underwent total TVM surgery (Table 3(c)). Mesh erosion took at least 6 M to become apparent in patients who underwent TVM surgery (Table 3(d)).

The POP recurrence rate after NTR and LSC procedures was also reviewed, even though these procedures were performed less often than TVM surgery. Only 1 of the 31 patients in the NTR group had recurrence. This patient was 75 years old and had a stage II uterine prolapse. A POP-Q stage II cystocele was noticed in this patient at a routine visit 6 months after the initial NTR. She did not undergo a second operation and continues to be followed up without any medical intervention. Four women had recurrence after LSC; 1 of these women had POP-Q stage II before surgery and 3 had stage III (Table 4). There was no statistically significant association between POP-Q stage and recurrence rate after LSC (Table 4(a)). The recurrence was noticed 6 months postoperatively in 2 cases, at 3 months in 1 case, and at 1 year in the remaining case (Table 4(b)). Only 1 of these 4 women underwent anterior and posterior TVM surgery because of an intolerable dragging sensation.

## 4. Discussion

In this retrospective study, we characterized surgical practice for POP in terms of recurrence rate and mesh erosion. Like most gynecologists in Japan, we have traditionally performed NTR without apical suspension (VH, anterior and posterior colpoplasty, or circumferential suturing of the levator ani muscles) but started performing TVM surgery in 2009 because of its reportedly favorable cure rate and low complication rate [14]. During this time, we have also performed NTR with SSLF in selected cases because we found that vaginal vault prolapse was common in patients who underwent NTR without apical suspension at our institution. We started performing LSC after the FDA warning about the use of TVM in 2011 [12]. Patients were selected for LSC on the basis of age and whether or not they were sexually active. Therefore, of the 308 patients who were treated surgically for POP during the study period, the majority underwent TVM surgery, which was our first-line treatment during the study period, and the remaining women underwent NTR or LSC; younger women were more likely to undergo LSC.

Although stage II POP was relatively common in our NTR group, there was no relationship between the type of surgery performed and the preoperative severity of POP or the recurrence rate during our study period. Furthermore, although there were no cases of mesh erosion after LSC, statistical evaluation showed that the rate of mesh erosion was not different between the TVM surgery group and the LSC group (both of which involve use of mesh). In addition, the reoperation rates for mesh erosion and/or recurrence of POP were not significantly different between the TVM surgery, NTR, and LSC groups, suggesting that these three procedures have comparable surgical outcomes.

Regarding outcomes of TVM surgery, POP recurrence rate was 6.17% at our institution over an 8-year period and is similar to that reported by Caquant et al. (6.9% during 6–18 months of follow-up) [14] and Sho et al. (7.0% during a median follow-up of 35.9 months) [15]. Moreover, as previously reported [16], we found that only severe prolapse before surgery was associated with anatomic failure postoperatively.

In a study reported by Achtari et al. in 2005, patient age was identified as a risk factor for mesh erosion [17], and Deffieux et al. subsequently reported that age over 70 years was an independent predictor [18]. Furthermore, Kaufman reported that age and sexual activity were risk factors for mesh erosion [19]. These reports indicated that the risk of mesh erosion may be highest at the extremes of life. However, in our study, there was no age-related pattern to the likelihood of mesh erosion. This finding is consistent with that in a recent report by Niu et al. [20], where no significant difference in age was found between their groups with and without mesh exposure. Furthermore, our experience was that mesh erosion was significantly more likely to occur after total TVM surgery. This finding suggests that previous hysterectomy could be one of the risk factors for postoperative mesh erosion after TVM surgery. In this connection, a previous report showed that vaginal sling procedures for urinary incontinence and concomitant hysterectomy were predictors of mesh erosion [21]. Cigarette smoking, operative technique, the type of mesh used, and presence of diabetes have also been reported to be predictors of mesh erosion [12, 22].

Although most of our POP repairs in the past 9 years were performed using TVM, the number of TVM procedures performed at our institution has steadily decreased after the US FDA warnings. Instead, we have started using LSC and NTR with SSLF for POP as often as possible. Although we have performed relatively small numbers of these operations, the surgical outcomes have been generally satisfactory. There is a growing tendency to avoid TVM surgery after the FDA alerts and the recent large cohort studies, all of which caution against vaginal repair with mesh [23, 24]. It is also true that postoperative stress urinary incontinence is often occurred in the patients who underwent TVM surgery [25, 26]. Nevertheless, we believe TVM surgery can still be effective with outcomes similar to those of the two alternative methods. In this study, we compared postoperative outcomes of three surgical methods for POP repair focused on recurrence rate and mesh erosion. Our study suggested that TVM surgery, NTR, and LSC have comparable outcomes; however, the outcomes of each technique need to be carefully evaluated over a long period of time, and the choice of procedure should be chosen on a case-by-case basis considering the condition of each patient.

## Figures and Tables

**Table 1:** Surgical procedures and postoperative outcomes.

Surgery		TVM surgery	NTR	LSC	*P*
*N*		243	31	34	
Age (years)		69.61 ± 8.31 (46–88)	65.68 ± 11.34 (35–85)	65.32 ± 3.23 (44–70)	*P* < 0.01^*a*^
POP-Q stage	II	45 (18.5%)	14 (45.2%)	9 (24.5%)	*P* < 0.01^*b*^
	III	168 (69.1%)	11 (35.5%)	24 (70.6%)	
	IV	30 (12.3%)	6 (19.4%)	1 (0.03%)	
Recurrence (%)		15 (6.17％)	1 (3.23)	4 (11.76％)	NS
Mesh erosion (%)		13 (5.35％)	N/A	0	NS
Reoperation (%)		5 (2.14％)	0 (0％)	1 (2.94％)	NS
		(Erosion, *n* = 3. Recurrence, *n* = 2)		(Recurrence)	

^*a*^ANOVA. ^*b*^Chi-squared test. NS: not statistically significant; TVM: tension-free vaginal mesh; NTR: native tissue repair; LSC: laparoscopic sacrocolpopexy.

**Table 2:** Cases of recurrence after TVM surgery.

(a) Age^*∗*^			
Age (years)	Recurrence, *n*	Total, *n*	Recurrence, (%)

41–50	0	2	0
51–60	1	32	1.13
61–70	9	92	9.78
71–80	5	100	5
81–90	0	17	0
	15	243	

(b) POP stage^#^			
Stage	Recurrence, *n*	Total, *n*	Recurrence, (%)

II	0	45	0
III	8	168	4.77
IV	7	30	23.33
	15	243	

(c) Type of surgery^*∗∗*^			
Surgical type	Recurrence, *n*	Total, *n*	Recurrence, %

TVM-A	2	49	4.08
TVM-AP	10	145	6.9
TVM-P	1	1	100
T-TVM	1	42	2.38
E-TVM	1	6	16.67
	15	243	

(d) Recurrence after initial surgery			
Month	Cases	Proportion of all cases (%)	

3	3	20	
6	2	13.33	
12	5	33.33	
24	3	20.00	
36	2	13.33	
	15	100.00	

^*∗*^No statistically significant differences were found between each age group. ^#^*P* < 0.01, the chi-squared test. ^*∗∗*^No statistically significant differences were found.

**Table 3:** Cases of mesh erosion after TVM surgery.

(a) Age^*∗*^			
Age (years)	Mesh erosion	Total	% of erosion

41–50	1	2	50
51–60	4	32	12.5
61–70	1	92	1.1
71–80	7	100	7
81–90	0	17	0

(b) Stage^#^			
Stage	Mesh	Total	% of erosion

II	3	45	6.67
III	7	168	4.17
IV	3	30	10
	13	243	

(c) Types of surgery^*∗∗*^			
Surgical type erosion	Mesh erosion	Total	% of erosion

TVM-A	2	49	4.08
TVM-AP	5	149	3.45
TVM-P	0	1	0
T-TVM	6	42	14.29
E-TVM	0	6	0
	13	243	

(d) Mesh erosion after initial surgery			
Months	Cases	% of total cases	

3 M	0	0	
6 M	3	20.00	
12 M	4	26.67	
24 M	5	33.33	
36 M	1	6.67	
FIG	13	100.00	

^*∗*^No statistically significant differences were found between each age group. ^#^No statistically significant differences were found. ^*∗∗*^*P* < 0.01, the chi-squared test.

**Table 4:** Recurrence of POP after LSC.

(a) Stage^*∗*^			
Stage	Recurrence	Total	%
II	1	9	11.1
III	3	24	12.5
IV	0	1	0
	4	34	
(b) Stage			
Months	Cases	% of total cases	
3M	1	25	
6M	2	50	
12M	1	25	

^*∗*^No statistically significant differences found.

## Data Availability

The clinical data used to support the findings of this study are available from the corresponding author upon request.
